# Isolation and Characterization of Three *Chalcone Synthase Genes* in Pecan (*Carya illinoinensis*)

**DOI:** 10.3390/biom9060236

**Published:** 2019-06-18

**Authors:** Chengcai Zhang, Xiaohua Yao, Huadong Ren, Kailiang Wang, Jun Chang

**Affiliations:** Research Institute of Subtropical Forestry, Chinese Academy of Forestry, Hangzhou 311400, China; c.c.zhang@caf.ac.cn (C.Z.); renhd@163.com (H.R.); wangkl163@163.com (K.W.); ylchj163@163.com (J.C.)

**Keywords:** pecan, *Carya illinoinensis*, phenolics, flavonoid, chalcone synthase, CHS, correlation analysis

## Abstract

Phenolics are a group of important plant secondary metabolites that have been proven to possess remarkable antioxidant activity and to be beneficial for human health. Pecan nuts are an excellent source of dietary phenolics. In recent years, many studies have focused on the separation and biochemical analysis of pecan phenolics, but the molecular mechanisms of phenolic metabolism in pecans have not been fully elucidated, which significantly hinders quality breeding research for this plant. Chalcone synthase (CHS) plays crucial roles in phenolic biosynthesis. In this study, three *Carya illinoinensis* *CHSs* (*CiCHS1*, *CiCHS2,* and *CiCHS3*), were isolated and analyzed. *CiCHS2* and *CiCHS3* present high expression levels in different tissues, and they are also highly expressed at the initial developmental stages of kernels in three pecan genotypes. A correlation analysis was performed between the phenolic content and *CHSs* expression values during kernel development. The results indicated that the expression variations of *CiCHS2* and *CiCHS3* are significantly related to changes in total phenolic content. Therefore, *CiCHSs* play crucial roles in phenolic components synthesis in pecan. We believe that the isolation of *CiCHSs* is helpful for understanding phenolic metabolism in *C. illinoinensis*, which will improve quality breeding and resistance breeding studies in this plant.

## 1. Introduction

Pecan (*Carya illinoinensis* (Wangenh.) K. Koch) belongs to the Juglandaceae family and is a commercially important nut crop native to North America that is cultivated throughout the world [1]. In recent years, tree nuts have been shown to be rich in unsaturated fatty acids and phenolic compounds, which present high antioxidant activity and are good for health. Many epidemiological studies have shown that high intake of tree nuts, fruits, and vegetables reduces the incidence of diabetes, cardiovascular diseases, cancer, and obesity [2,3]. Among different plant active ingredients, phenolics are crucial for these potential health benefits [2]. Compared to other nut types, pecan was reported to have higher content of phenolics, flavonoids, and condensed tannin and has been recommended as an excellent source of diet phenolics [4,5]. Thus, many studies have focused on the separation of phenolics and characterization of antioxidant capacity of different components in pecan [6,7,8,9]. However, the molecular basis of phenolic biosynthesis in pecan has not been fully elucidated [10]. Recently, an RNA-Seq dataset of pecan kernels was released, and in total, 36 putative structural genes involved in flavonoid synthesis were reported [10]. However, until now, no flavonoid related gene has been identified or characterized in *C. illinoinensis*.

Chalcone synthase (CHS) is the first committed enzyme in flavonoid synthesis, which channels the flow of the “phenylpropanoid pathway” toward the “flavonoid pathway”. This enzyme plays an essential role in many physiological processes, including fruit/seed development, floral pigmentation, pollination, and resistance to biotic and abiotic stresses in plants [11]. Therefore, it has been widely studied in various plants. For example, knockout or overexpression of *CHS* has been shown to influence the synthesis and accumulation of phenolics in walnut [12], maize [13], *Silybum marianum* [14], flax [15], and *Arabidopsis thaliana* [16]. Moreover, *CHS* has been proved to regulate floral color in *Petunia hybrida* [17], seed coat color in soybean [18], verticillium disease resistance in both *A. thaliana* and cotton [19], dicamba resistance in *Kochia scoparia* [20], antioxidant capacity [21] and cell wall components in flax [22]. In addition, allelic variations present in *CHS* might affect the expression level or catalytic activity of the enzyme and can be converted into functional markers for molecular marker assisted selection (MAS) in plants such as *Zingiber officinale* [23] and Indian mulberry [24]. Genus *Carya* includes 18 species [25], among them pecan and 3 types of Cathay hickory (*C. cathayensis*, *C. hunanensis*, and *C. dabieshanensis*) have economic values. Nevertheless, *CHSs* in *Carya* have not been cloned, which significantly hinders the study of phenolic metabolism and biotic/abiotic stress tolerance research in these species.

In the present study, the content variation of total phenolics (TPC), total flavonoids (TFC) and condensed tannins (CTC) during kernel development was detected in three pecan genotypes. The full-length cDNAs of 3 *C. illinoinensis CHSs* (*CiCHSs*) were cloned and analyzed. Subsequently, a quantitative real time PCR (qRT-PCR) method was employed to determine the expression of CiCHSs in six types of tissues and seven different developmental stages of embryo. Finally, correlation analyses between the phenolic content and the gene expression patterns were performed. We believe that the cloning and characterization of *CiCHSs* will help to reveal the genetic basis of phenolic synthesis and accelerate MAS breeding studies and stress tolerance research in *C. illinoinensis* as well as in other species in the genus *Carya*.

## 2. Results and Discussion

### 2.1. Total Phenolic, Total Flavonoid, and Condensed Tannin Content

**Figure 1:**
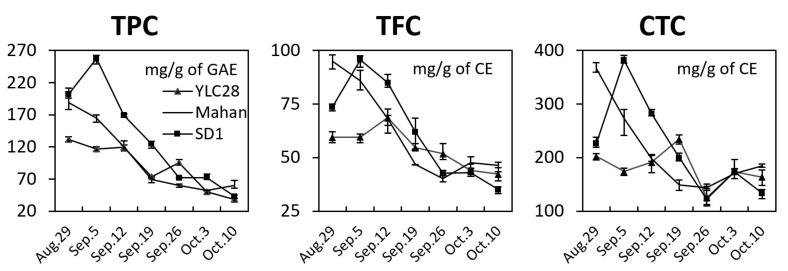
The dynamic changes of phenolic components content during pecan kernel development in three genotypes. Total phenolic content (TPC), total flavonoid content (TFC), and condensed tannin content (CTC). The experiments were repeated three times and the data are presented as the mean ± standard error (SE).

To investigate the dynamic changes of phenolic components during pecan kernel development, the TPC, TFC, and CTC in seven developmental stages of embryos from “Mahan”, “SD1”, and “YLC28” were analyzed (Figure 1). Of these, the phenolic content of four time points (August 29, September 12, September 26 and October 10) of “YLC28” came from our previous study [10]. The highest TPCs of “Mahan” (188.89 mg/g GAE) and “YLC28” (131.70 mg/g GAE) were seen at the first stage, and TPCs gradually decreased thereafter. However, TPC in “SD1” first increased and reached a peak in September 5 (257.28 mg/g GAE), then decreased during embryo ripening. Similarly, Amin et al. (2017) reported that TPC decreased during walnut kernel ripening [26]. The changes of TFC in “Mahan” were similar to those of TPC in this cultivar. TFC in both “YLC28” and “SD1” increased first and then decreased, but the highest TFC in “SD1” (95.63 mg/g CE) and “YLC28” (68.61 mg/g CE) occurred at the second and the third developmental stages, respectively. CTC in “Mahan” decreased from August 29 to September 26 and exhibited a slight increase thereafter. In “SD1”, CTC sharply increased from August 29 to September 5 (381.56 mg/g CE), then decreased and reached a minimum level on September 26 (123.01 mg/g CE), after which it increased slightly. CTC in “YLC28” showed a different variation trend from the other 2 genotypes, with a slow increase during the early developmental period, and showed the highest content on September 19 (234.37 mg/g CE), and then shared similar trends with “SD1”. These results indicated that the phenolic profiles of different genotypes were significantly different. Similar results were also reported by Jia et al. (2018) who detected dynamic changes of phenolics during pecan kernel ripening in 5 cultivars. They found that TPC was highest at the early stage of kernel development and declined quickly thereafter. TFC and CTC exhibited similar profiles but were slightly different among different cultivars [6].

### 2.2. Cloning and Analysis of Three CiCHSs

**Figure 2:**
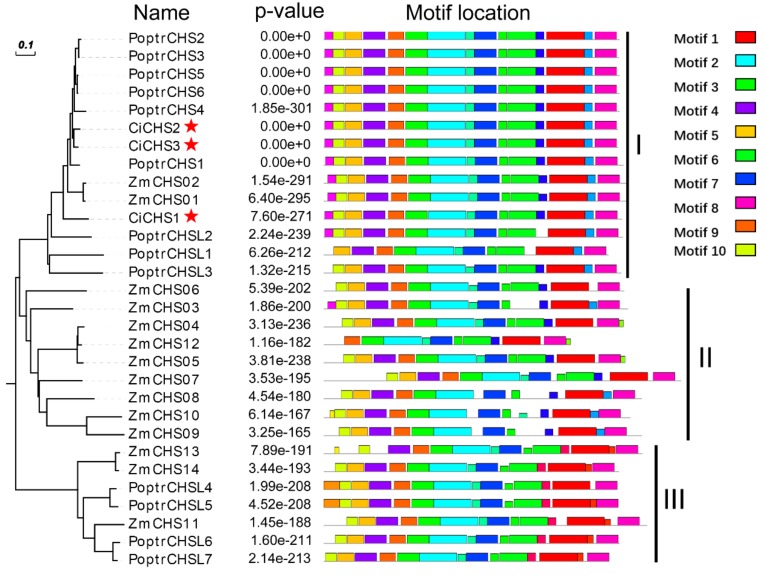
Phylogenetic dendrogram of three amino acid sequences of CiCHSs and CHSs from maize (ZmCHS01~14) and *Populus* (PoptrCHS1~6, PoptrCHSL1~7) constructed using the neighbor-joining method (1000 bootstrap replications). The conserved motifs in each protein sequence were predicted using the Motif Elicitation (MEME) web server. The red stars show the positions of the three CiCHSs.

Three putative *CHSs* were selected from our previous study on transcriptomic profiles of pecan kernels in four developmental stages [10]. The target bands were obtained and sequenced (Figure S1, Figure S2). The resulting full-length cDNA sequences of three *CiCHSs* of strain “YLC28” were obtained and submitted to GenBank with new gene IDs (Table 1). The three *CiCHSs* were translated into amino acid sequences, and we constructed a dendrogram with these sequences and with CHS protein sequences from maize [27] and *Populus* [28] (Figure 2). The dendrogram revealed that the sequences were classified into three clusters (clusters I, II, and III). Most CHSs (14 out of 30) were classified into group I, including three CiCHSs, nine PoptrCHSs, and two ZmCHSs. In addition, nine ZmCHSs were clustered into group II, and three ZmCHSs and four PoptrCHSLs were clustered into group III. The conserved motifs and domains of each CHS were investigated using MEME and SMART programs, respectively. Ten conserved motifs (namely, motifs 1–10) and two domains of “Chal_sti_synt_N” and “Chal_sti_synt_C” were identified (Figure 2). This result was consistent with the findings of a previous study on rice [29]. The “Chal_sti_synt_N” domain was represented by motifs 2, 4–7, 9, and 10, while domain “Chal_sti_synt_C” was represented by motifs 1, 3, and 8. The two domains of “Chal_sti_synt_N” and “Chal_sti_synt_C” were highly conserved in three CiCHSs. *CHSs* constitute a large gene family in the plant genome [27,28,29] and perform different functions in plant development and stress response. Therefore, the functional identification of *CiCHSs* in pecan should be performed in the future.

**Figure 3:**
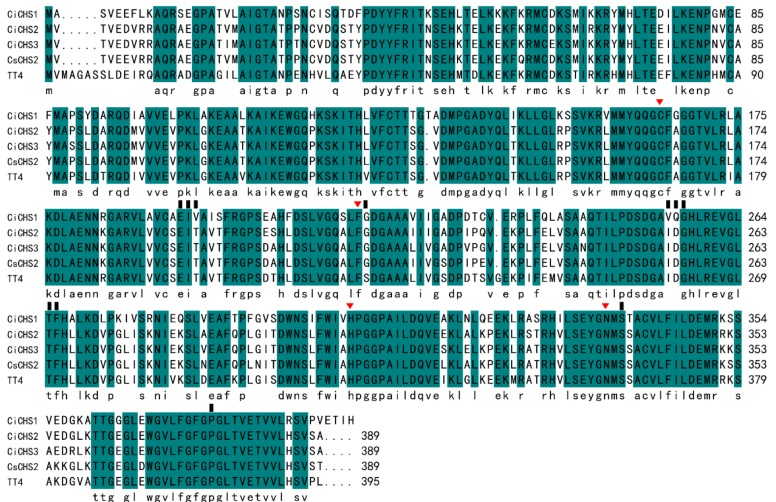
Multiple alignment of five CHSs: CiCHS1, CiCHS2, CiCHS3, TT4 (AT5G13930), and CsCHS2 (P48387.1). The red triangles indicate the positions of active sites. The black squares indicate the positions of product binding sites.

A multiple sequence alignment of TT4 (AT5G13930), CsCHS2 (P48387.1), CiCHS1, CiCHS2, and CiCHS3 was constructed by using DNAMAN software (Figure 3). CiCHS1 shared 73.25% and 74.75% identity with TT4 and CsCHS2, respectively. For CiCHS2, the identity values were 82.75% (with TT4) and 92.50% (with CsCHS2). In addition, for CiCHS3, the values were 83.00% (with TT4) and 91.75% (with CsCHS2). *TT4* encodes a CHS in *Arabidopsis*, where it regulates flavonoid accumulation and affects plant development and the gravitropism of roots [30]. Tea accumulates much polyphenol in leaves, and CsCHS acts as a rate-limiting enzyme in the flavonoid pathway [31]. Therefore, the high identity of the 3 CiCHSs with TT4 and CsCHS2 implies that they have crucial roles in phenolic synthesis.

### 2.3. Expression Profiles of 3 CiCHSs in Different Tissues

**Figure 4:**
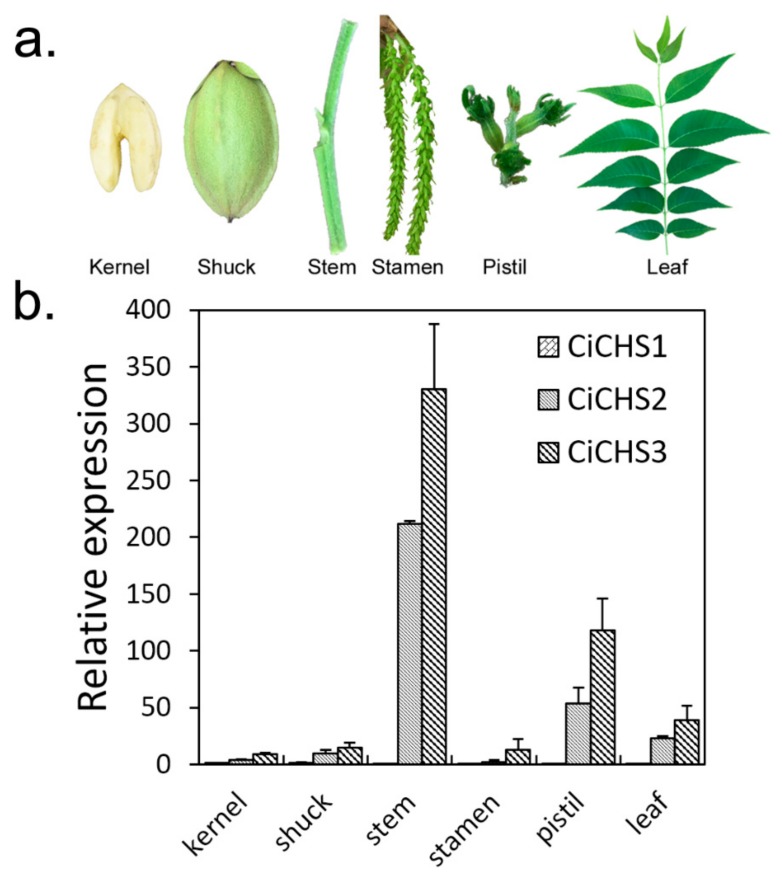
Six types of tissue (**a**), and the expression patterns of the three *CiCHSs* in the six different tissues (**b**). Each reaction was performed in three biological replicates, and the data are presented as the mean ± standard error (SE).

The expression patterns of the *CiCHSs* in various tissues were analyzed using qRT-PCR (Figure 4). As shown in Figure 4b, *CiCHS1* was mainly expressed in shuck but exhibited the lowest expression level in different tissues among the three *CiCHSs*. *CiCHS2* and *CiCHS3* showed high expression levels in different tissues and shared similar expression profiles with each other. Both *CiCHS2* and *CiCHS3* had the highest expression in tender stem and showed high transcript levels in pistil and leaf. In recent years, phenolics have been isolated and identified from pecan kernel, leaves, bark, and shells [7,9,32,33,34]. The antioxidation and potential health efficacy of different tissue-derived phenolics in pecan were also determined [32,33]. Moreover, pecan shell extracts can inhibit tumor cell growth and may be potential candidates for cancer therapy [34]. In addition, phenolic components and flavonoids are associated with biotic/abiotic stress tolerance and the fertilization process [11]. The high expression levels of *CiCHSs* in stem, pistils, and leaves indicate that phenolic synthesis might be active in these tissues. Mo et al. (2017) reported that the CHS protein was differentially expressed in the graft union of pecan at five developmental stages after grafting. The CHS might relate to the callus proliferation by regulating the accumulation of flavonoids after grafting [35]. Similarly, *PtrCHS4* was mainly expressed in leaves and stems in *Populus trichocarpa*, and its expression was stimulated by wounding treatment [36]. Therefore, the *CiCHSs* might realize different functions through the regulation of flavonoid biosynthesis in different organs. In addition, the higher transcript levels of *CiCHS2* and *CiCHS3* in kernels implies that these two genes might be associated with phenolic accumulation in pecan kernels. In addition, the higher transcript levels of *CiCHS2* and *CiCHS3* in kernels imply that the two genes might associated with phenolic accumulation in pecan kernels.

### 2.4. Expression Profiles of 3 CiCHSs during Pecan Kernel Development

**Figure 5:**
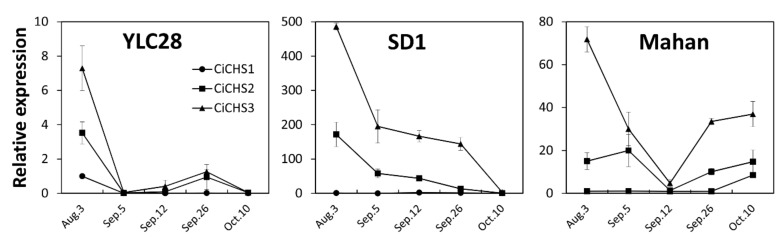
The expression profiles of the three *CiCHSs* during kernel maturation in three pecan genotypes. Each reaction was performed in three biological replicates, and the data are presented as the mean ± standard error (SE).

To investigate gene expression variation during kernel development, the transcript profiles of the three *CiCHSs* were identified in three pecan genotypes (Figure 5). The transcript levels were mostly downregulated during embryo development and ripening. *CiCHS3* showed the highest expression amount at the early developmental stage of pecan kernels among the three genotypes. *CiCHS2* shared a similar expression pattern with *CiCHS3* even though its transcript level was lower. *CiCHS1* presented the lowest expression values during the pecan kernel development period. These results were in accordance with the tissue expression patterns of the three *CiCHSs*. In addition, the *CiCHSs* presented obviously different expression behavior among the three pecan genotypes. In ‘YSL28′, the expression of *CiCHS3* and *CiCHS2* were sharply decreased from August 29 to September 5, then showed a slight increase on September 26 and were downregulated thereafter. Interestingly, the transcript profiles were similar to TPC variation during the kernel development of “YLC28”. For “SD1”, the expression of *CiCHS3* and *CiCHS2* were also decreased during embryo ripening, however, the decrease trend gradually flattened from September 5 to September 26. *CiCHS3* and *CiCHS2* in “Mahan” were highly expressed during the initial developmental period of kernels and presented the lowest expression levels on September 12, and then were upregulated thereafter. Therefore, the different phenolic profiles of the three pecan genotypes might result from different expression patterns of *CiCHSs*. On the other hand, the expression levels of *CiCHS2* and *CiCHS3* were obviously different in the three genotypes. Trojan et al. (2014) reported that the different expression levels of CHS may be associated with the different contents of delphinidin-3-glucoside and cyanidin-3-glucoside in three genotypes of wheat [37]. Because only small differences among TPC, TFC, and CTC were observed compared to the gene expression levels between the three pecan genotypes; the differentially expressed *CiCHSs* may be related to the different contents of specific metabolites in the three pecan genotypes. This possibility should be investigated in the future.

### 2.5. Correlation Analysis

**Figure 6:**
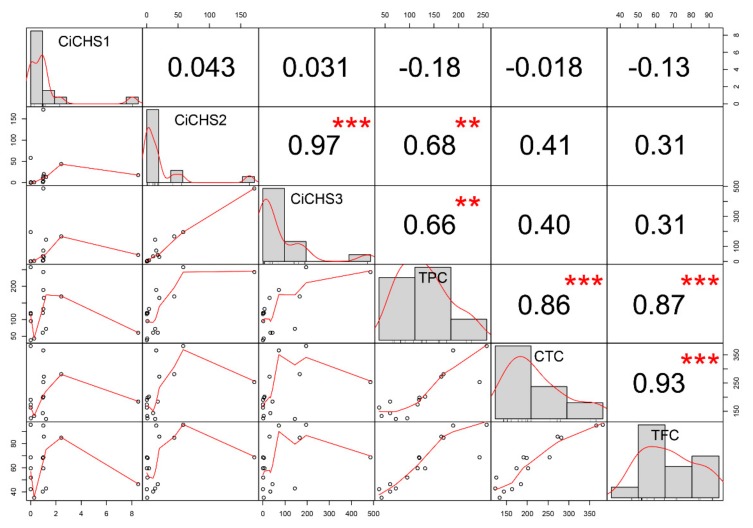
Correlation coefficient of phenolic content and gene expression during pecan kernel development. The distribution of gene expression values and phenolics component contents are shown on the diagonal. To the bottom left are the bivariate scatter plots with best fit lines displayed. Correlation coefficients are shown above the diagonal. “***” and “**” denote significance with *p* values of 0.001 and 0.01, respectively.

Correlation analysis was performed between the phenolic contents and the gene expression values across pecan kernels development to determine the contribution of each *CiCHS* to the phenolic components of TPC, CTC, and TFC. *CiCHS1* showed no significant correlation with TPC, CTC, and TFC (Figure 6). In contrast, high correlation coefficients were observed between CiCHS2 and CiCHS3 and TPC, 0.68 (*p* ≤ 0.01) and 0.66 (*p* ≤ 0.01), respectively. The correlation between phenolic accumulation and *CHS* expression was also confirmed in other species. For example, in walnut, a large positive relationship was noticed between flavonoid content and *CHS* expression during rhizogenesis, and weak positive relationships were observed between flavonol or proanthocyanidin content and *CHS* expression [38]. In *Brunfelsia acuminata*, the expression of *BaCHS* was positively correlated with petal anthocyanin contents [39]. In a maize cultivar, ‘SW93′ (purple kernels), two *CHSs* were upregulated along the kernel as the color deepened and anthocyanins accumulated [40]. In *C. sinensis*, high *CHS* transcript values were associated with a high amount of total tea polyphenols and total catechin [41,42]. However, lower correlation coefficients indicating no significant correlation were observed between *CiCHS2* and CTC (0.41), *CiCHS2* and TFC (0.31), *CiCHS3* and CTC (0.40), and *CiCHS3* and TFC (0.31). These results might be caused by the downstream genes of the flavonoid biosynthesis pathway that together regulate the synthesis of flavonoids and condensed tannin in pecan kernels, and these genes might play more important roles in determining the content and composition of flavonoids. In addition, flavonoids may transport from synthetic sites to other parts of the plant [43,44]. The redistribution of flavonoids between organs and tissues might also occur in pecan, which would thus influence the concentration of flavonoids in kernels.

## 3. Materials and Methods

### 3.1. Plant Materials, RNA Isolation, and Reverse Transcription

Seven different developmental stages (29 August, 5 September, 12 September, 19 September, 26 September, 1 October, and 10 October) of embryo were collected from each of three pecan genotypes, “Mahan” (an important cultivar introduced from USA), “YLC28” (an excellent strain), and “SD1” (a seedling tree) in 2017. Tissues of leaves, shuck, young stem, and male and female flowers were collected from “YLC28”. The samples were frozen rapidly in liquid nitrogen and stored at −80 °C for further analysis. Samples were collected in triplicate. The three pecan genotypes used in this study were planted at Jiande (29° N, 119° W), China. Total RNA was extracted from different tissues using the RNAprep Pure Plant Kit (Tiangen, Beijing, China). The quality of the RNA was confirmed by a Quawell Q5000 spectrophotometer (Quawell Technology, Inc., San Jose, CA, USA) and electrophoresis through a 1% agarose gel. One microgram of the total RNA was reverse-transcribed into cDNA using the PrimeScript 1st Strand cDNA Synthesis Kit (Takara, Dalian, China).

### 3.2. TPC, TFC, and CTC Detection

The samples of different developmental stages of kernels were dried using a freeze dryer (FreeZone, Labconco Corp., Kansas City, MO, USA) for 96 h. The kernels were fully grinded and then defatted. The phenolic compounds extraction and the detection of TPC, TFC, and CTC were performed as described previously [7]. A similar analysis was also performed in our previous RNASeq paper [10]. The TPC was measured by the Folin–Ciocalteu method, and gallic acid equivalents (GAE) were used as the standard measurements. The TFC was detected using a 723N spectrophotometer (Shanghai Precision and Scientific Instrument Co. Ltd., Shanghai, China) at a wavelength of 510 nm, and the results were expressed in catechin equivalents (CE). The CTC was detected by a 723N spectrophotometer at a wavelength of 500 nm, and the results were expressed in CE.

### 3.3. Cloning and Bioinformatics Analysis of CiCHSs

Three unigenes with strong homology to chalcone synthase genes were selected from our previous study [10]. In that study, the transcriptomic profiles of pecan kernels (from a strain of “YLC28”) in four developmental stages were analyzed. In total, 36 differentially expressed putative flavonoid-related structural genes were isolated, including three phenylalanine ammonia-lyases (*PALs*), three *CHSs*, two chalcone-flavanone isomerases (*CHIs*), one flavanone 3-hydroxylase (*F3H*), two flavonoid 3′-hydroxylases (*F3′Hs*), two flavonoid 3′,5′-hydroxylases (*F3′5′Hs*), one dihydroflavonol 4-reductase (*DFR*), one anthocyanidin synthase (*ANS*), two leucoanthocyanidin reductases (*LARs*), and two anthocyanidin reductases (*ANRs*). The ORFs of the putative *CHSs* were predicted using a DNAstar software Editseq program (DNASTAR, Inc., Madison, WI). Respective gene specific primers were designed based on the 5′-UTR and 3′-UTR of the three genes, using Primer 5.0 software (Premier Biosoft International, Palo Alto, CA, USA). The cDNA derived from “YLC28” kernels was used to clone full-length cDNAs of the *CiCHSs*. PCRs were performed using the KOD FX polymerase (Toyobo, Osaka, Japan). The 50 μL reaction volume contained 1 μL KOD FX polymerase, 25 μL 2 × PCR buffer for KOD FX, 10 μL dNTP (2.0 mM), 0.75 μL each primer (100 μM), 500 ng cDNA and ddH_2_O up to 50 μL. The PCR program was as follows: an initial denaturation step at 94 °C for 2 min, followed by 35 cycles of 98 °C for 10 s, 56 °C for 30 s and 68 °C for 1 min, and a final extension at 68 °C for 7 min. PCR products were electrophoresed on a 1% agarose gel, stained with GenGreen solution and purified using a TIANgel Midi Purification Kit (Tiangen, Beijing, China). Then, the target fragments were inserted into the pMD18-T vector (TaKaRa, Dalian, China) and transformed into *Escherichia coli* DH5α cells. Positive recombinant plasmids were sequenced using M13 primers in both directions. After that, the full-length cDNAs of the *CiCHSs* were obtained after aligning the sequencing results to the target unigenes. The open reading frames (ORFs) and amino acid sequences of each gene were predicted using the EditSeq program (DNASTAR, Inc., Madison, WI, USA). Theoretical molecular weight (MW) and isoelectronic point (pI) were calculated using the ProtParam tool (http://web.expasy.org/protparam/). A multiple sequence alignment was generated using DNAMAN software (www.lynnon.com). Conserved domains of the CiCHSs were predicted using InterProScan (http://www.ebi.ac.uk/Tools/pfa/iprscan/). Because maize and *Populus* are excellent model species, the distributions of *CHSs* in their genomes have been fully investigated [27,28]. A phylogenetic tree was constructed using three *CiCHS*-derived amino acid sequences and CHSs from maize [28] and *Populus* [27] with ClustalX 2.0 (http://www.clustal.org/) and MEGA 5.0 software (www.megasoftware.net) using a neighbor-joining method with 1000 bootstrap replicates. The online software of MEME (http://meme-suite.org/tools/meme) and SMART (http://smart.embl-heidelberg.de/) was used to predict the conserved domains of each CHS.

### 3.4. Expression Patterns of CiCHSs

One microgram of total RNA of each sample, including seven different developmental stages of embryo and six different tissue types, were used for first-strand cDNA synthesis with a PrimeScript 1st Strand cDNA Synthesis Kit (Takara, Dalian, China). The primers of *CiCHS2* and *CiCHS3* came from our laboratory previously [10]. Gene-specific primers of CiCHS1 were designed using Primer 5.0 (Table S1). Expression profiles of *CiCHSs* were detected using qRT-PCR on a QuantStudio 7 Flex Real-Time PCR System (Applied Biosystems) and were normalized to the *18s RNA* housekeeping gene [45]. The qRT-PCR reactions were performed according to the PrimeScript RT reagent qPCR Kit manual (TaKaRa, Dalian, China). The 20 μL reaction volume contained 10 μL of 2 × SBYR Premix Ex Taq II, 0.4 μL of 50 × ROX reference Dye, 0.4 μL of each primer (10 μM) and 80 ng of cDNA. The PCR program was 95 °C for 30 s; 40 cycles of 95 °C for 5 s and 61 °C for 34 s; and a dissociation stage. The relative expression of the *CiCHSs* was calculated using the 2^−ΔΔCt^ method. Each reaction was performed in 3 biological replicates. The expression patterns of *CiCHS2* and *CiCHS3* at four time points (August 29, September 12, September 26, and October 10) of “YLC28” were analyzed in our previous study [10].

### 3.5. Correlation Analysis

The correlation analysis between the change in phenolic content and gene expression patterns was performed using R package ‘Hmisc’ (http://CRAN.R-project.org/package=Hmisc) and ‘PerformanceAnalytics’ (http://CRAN.R-project.org/package=PerformanceAnalytics).

## 4. Conclusions

Three pecan *CiCHS* genes were isolated and characterized for the first time in this study. The full-length cDNA of the three *CiCHSs* were 1258 bp to 1432 bp, the predicted amino acids ranged from 389 to 394. The three CiCHSs shared two conserved domains of “Chal_sti_synt_N” and “Chal_sti_synt_C” with CHSs from maize and *Populus*. Multiple alignment analysis confirmed that CiCHSs share much of their identity with TT4 and CsCHS. The expression levels of *CiCHS2* and *CiCHS3* in various pecan tissues were dramatically higher than that of *CiCHS1*. *CiCHS2* and *CiCHS3* exhibited the highest expression levels in the initial development stages of kernels, and were differentially expressed across the kernel development. However, slight expression changes were observed for *CiCHS1*. These findings indicated that *CiCHS2* and *CiCHS3* might be associated with the accumulation of phenolic components in pecan kernels, and this information will promote quality breeding in this plant. High correlation coefficients were observed between the expression values of *CiCHS2* and *CiCHS3* and TPC. These findings indicated that *CiCHS2* and *CiCHS3* might be associated with phenolic components accumulation in pecan kernels, and this information will promote quality breeding in this plant.

## Figures and Tables

**Table 1:** Information regarding 3 *CiCHSs*.

Gene	Full-Length cDNA (bp)	ORF (bp)	Amino Acids No.	Molecular Weight (kDa)	Isoelectric Point (pI)	Accession No.
*CiCHS1*	1432	1185	394	43.09	6.23	MK690794
*CiCHS2*	1309	1170	389	42.64	6.12	MK690795
*CiCHS3*	1258	1170	389	42.59	6.34	MK690796

## Supplementary Materials

The following are available online at https://www.mdpi.com/2218-273X/9/6/236/s1, Table S1. Specific primers used in this study, Figure S1. The target PCR products of three full-length cDNA of *CiCHSs*, Figure S2. Full-length cDNA sequences of 3 *CiCHSs*.
