# Supplementary material for: Isolation and Characterization of Three Chalcone Synthase Genes in Pecan (Carya illinoinensis)
Source: Biomolecules. 2019 Jun 18;9(6):236. doi: 10.3390/biom9060236 (PMC6627513; doi:10.3390/biom9060236)
Supplement: Supplementary file 1 [file biomolecules-09-00236-s001.zip › Table S1.docx]

**Table 1.** Specific primers used in this study.

| **Primer** | **Primer Sequence (5'-3')** | **Product Length (bp)** | **Use** |
| --- | --- | --- | --- |
| *CiCHS1* | Forward: ATTCTCCGTTTCCAACAGCC | 1432 | Gene clone |
|  | Reverse: ATTGGTTACAGATCCTGAGAGAGC |  |  |
| *CiCHS2* | Forward: TATTTTGGTAACCCCAGTTCTCTCC | 1309 | Gene clone |
|  | Reverse: GGCACAGACCTTAGAAACACCGT |  |  |
| *CiCHS3* | Forward: CTAAGTGCCCAACCTTTGAAGAGAT | 1258 | Gene clone |
|  | Reverse: CAAGTAAAAGAGAAACAGGGGAACC |  |  |
| *rtCHS1* | Forward: GACCCCGATACATGCGTTGA | 115 | qRT-PCR |
|  | Reverse: ATGTCAAGCCTACTTCGCGT |  |  |
| *rtCHS2* | Forward: ATGAGATGAGAAGGAAGTCTGTTGA | 135 | qRT-PCR |
|  | Reverse: GCCTCTAAGCAGACACACTATGC |  |  |
| *rtCHS3* | Forward: GGATGAGATGAGGAAGAAGTCGG | 154 | qRT-PCR |
|  | Reverse: GATAATGGGGGAGGAAGCTAATT |  |  |
| *18s* | Forward: ACATCTTACCACGATACATAAC | 134 | qRT-PCR |
|  | Reverse: AACTTGCGTTCAAAGACTC |  |  |
