# Supplementary figures and images for: Isolation and Characterization of Three Chalcone Synthase Genes in Pecan (Carya illinoinensis)
Source: Biomolecules. 2019 Jun 18;9(6):236. doi: 10.3390/biom9060236 (PMC6627513; doi:10.3390/biom9060236)

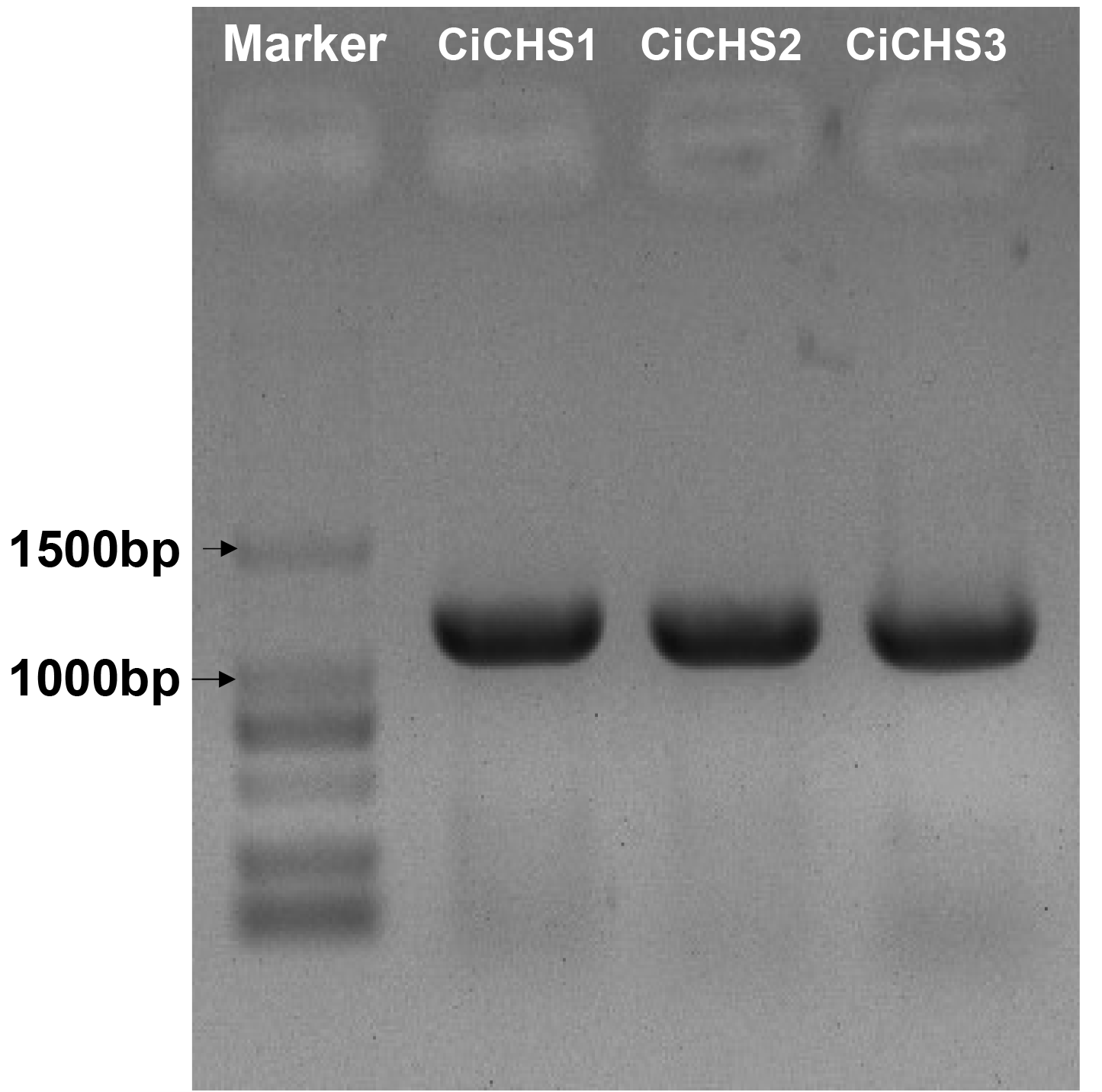

Supplement: Supplementary file 1 [file biomolecules-09-00236-s001.zip › Figure S1.tif]

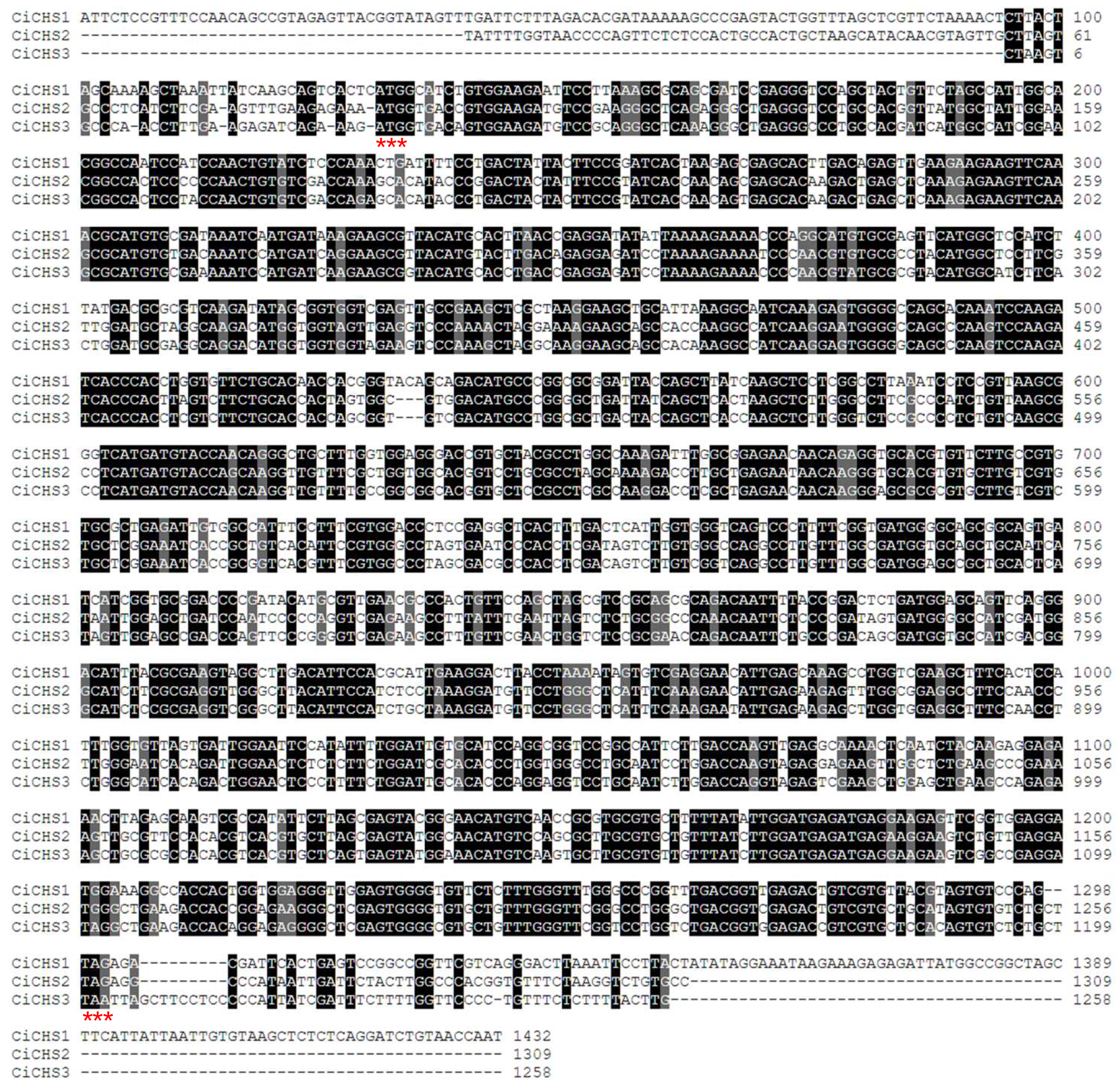

Supplement: Supplementary file 1 [file biomolecules-09-00236-s001.zip › Figure S2.tif]
